# The Interaction between Fluid Wall Shear Stress and Solid Circumferential Strain Affects Endothelial Gene Expression

**DOI:** 10.1371/journal.pone.0129952

**Published:** 2015-07-06

**Authors:** Ronny Amaya, Alexis Pierides, John M. Tarbell

**Affiliations:** Department of Biomedical Engineering, City College of New York, City University of New York, New York, New York, 10031, United States of America; Vanderbilt University Medical Center, UNITED STATES

## Abstract

Endothelial cells lining the walls of blood vessels are exposed simultaneously to wall shear stress (WSS) and circumferential stress (CS) that can be characterized by the temporal phase angle between WSS and CS (stress phase angle – SPA). Regions of the circulation with highly asynchronous hemodynamics (SPA close to -180°) such as coronary arteries are associated with the development of pathological conditions such as atherosclerosis and intimal hyperplasia whereas more synchronous regions (SPA closer to 0°) are spared of disease. The present study evaluates endothelial cell gene expression of 42 atherosclerosis-related genes under asynchronous hemodynamics (SPA=-180 °) and synchronous hemodynamics (SPA=0 °). This study used a novel bioreactor to investigate the cellular response of bovine aortic endothelial cells (BAECS) exposed to a combination of pulsatile WSS and CS at SPA=0 or SPA=-180. Using a PCR array of 42 genes, we determined that BAECS exposed to non-reversing sinusoidal WSS (10±10 dyne/cm^2^) and CS (4 ± 4 %) over a 7 hour testing period displayed 17 genes that were up regulated by SPA = -180 °, most of them pro-atherogenic, including NFκB and other NFκB target genes. The up regulation of NFκB p50/p105 and p65 by SPA =-180° was confirmed by Western blots and immunofluorescence staining demonstrating the nuclear translocation of NFκB p50/p105 and p65. These data suggest that asynchronous hemodynamics (SPA=-180 °) can elicit proatherogenic responses in endothelial cells compared to synchronous hemodynamics without shear stress reversal, indicating that SPA may be an important parameter characterizing arterial susceptibility to disease.

## Introduction

**Fig 1:**
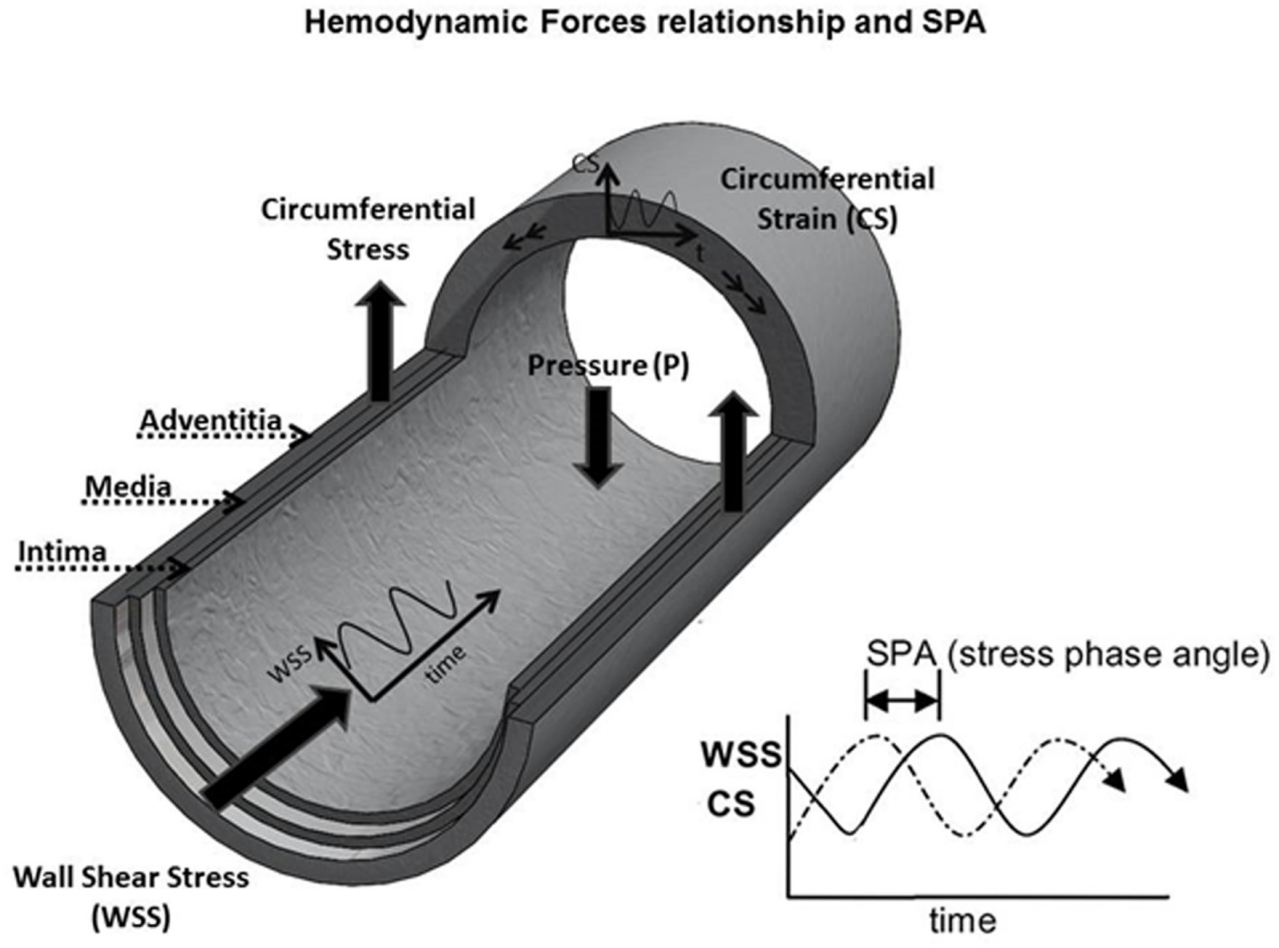
Simultaneous WSS and CS on EC are characterized by the SPA. Blood flow in the axial direction induces wall shear stress (WSS) and the changes in pressure during the cardiac cycle induce circumferential strain and circumferential stress (CS) on the EC lining the wall of the blood vessel. Due to impedance of the distal circulation, local inertial effects associated with flow in larger vessels and arterial geometry, there is a time lag between WSS and CS characterized by the stress phase angle—SPA = φ(CS-WSS)

The fluid wall shear stress (WSS) driven by pulsatile blood flow and the solid circumferential stress (CS) driven by pulsatile blood pressure and associated strain, act simultaneously on endothelial cells (EC) lining blood vessels modulating their biological activity. Due to distal impedance at a vascular site of interest (global effect) and the inertial effects of blood flow at the site (local effect), a time lag arises between CS and WSS that is referred to as the “Stress phase angle”—SPA [1] (Fig 1). The SPA has been shown to play a role in regulating the release of vasoactive molecules such as nitric oxide (NO), endothelin-1 (ET-1), and prostacyclin (PGI_2_) in vitro. The production of NO and PGI_2_ by bovine aortic endothelial cells (BAEC) were suppressed, while the production of the vasoconstrictor ET-1 was increased under highly asynchronous hemodynamic conditions compared with more synchronous hemodynamic conditions [1]. Recent computational and theoretical studies of SPA have been carried out for a coronary artery [2], the carotid bifurcation [3], end-to-end anastomosis [4], and the EC plasma membrane [5], showing that large negative SPA occurs in regions of the circulation where atherosclerosis and intimal hyperplasia are localized. In support of these findings, in vivo studies showed that coronary arteries with highly asynchronous hemodynamics exhibited a pathological gene expression pattern for a limited number of genes [6] and that intimal hyperplasia does not correlate with WSS alone in coronary arteries [7]. Other recent reviews and computational analyses further support the idea that WSS characteristics alone do not completely account for the localization of vascular disease [7–9]. These observations suggest that hemodynamic factors other than WSS may play a critical role in localization and development of atherosclerosis.

Most studies of fluid mechanics and atherosclerosis have used the time-average WSS and oscillatory shear index (OSI—an index of direction changes in WSS; it measures the extent to which the shear stress reverses its direction over the flow cycle, as the parameters that correlate the relationship between hemodynamics and atherosclerosis since the work of Ku et al. [10]. We believe the SPA should be included as an important hemodynamic parameter as well because it measures the degree of asynchrony between the main biomechanical forces acting on the EC (CS and WSS), combining solid mechanics and fluid mechanics.

To further support the hypothesis that SPA is an important parameter, we have used a novel hemodynamic simulator to compare gene expression profiles (39 target genes and 3 housekeeping genes) of BAECs under highly asynchronous, atheroprone hemodynamics (SPA = −180°) with atheroprotective, synchronous hemodynamics (SPA = 0°). BAECs were chosen since they have been utilized extensively as a model system for the study of mechanical effects on EC. A limited set of genes was investigated rather than a large array because the SPA is still not widely accepted as an important mechanical factor in EC mechano-stimulation. We observed that the expression of 17 out of 39 genes differed significantly after exposure to identical WSS and CS waveforms that differed only by the SPA (0° or -180°).

## Materials and Methods

Cell culture components obtained from Sigma (St. Louis, MO) include: bovine serum albumin (BSA, 30% solution), trypsin, penicillin streptomycin, MEM (phenol red free), sodium bicarbonate, fibronectin, fetal bovine serum (FBS), L-Glutamine. Dulbecco's PBS (1x without Ca2+ and Mg2+) from Fisher Scientific (Houston, TX).

BAECs were purchased from VEC Technologies (Rensselaer, NY) and grown in T-75 flasks with 10% FBS-MEM. Silicone sheets (Down Corning Corporation, MI) were used as the substrates for cell culture. BAECs were seeded onto the upper surface of silicone membranes of 0.020" thickness. First the membranes were laser cut to the geometrical specifications of the bioreactor, and then silicone substrates were washed for 20min with a gentle detergent and autoclaved for 30 minutes. A region delimited by an area of 1.5 x 4.5 cm on each substrate was coated using bovine plasma fibronectin (30μg/ml in MEM) for 1 hour at room temperature. The BAECs between passages 3 and 7 were plated at a density of 1.0x10^5^ cells/cm^2^ onto the treated silicone substrates. ECs were grown using with 10% FBS until confluency in a controlled environment (37C and 5% CO_2_ air). The EC monolayer reached confluence within four days.

### Gene expression

At the end of the stretch and shear experiments, RNA was isolated using TRIzol reagent (Invitrogen) following the manufacture’s protocol. RNA was purified using the RNeasy kit (Invitrogen), and then purified RNA was converted into cDNA by reverse transcription (RT). For analyzing gene expression, quantitative real-time PCR (RT-qPCR) was performed for 42 genes on the ABI PRISM 7000 sequence detection system (Applied Biosystems). Using the geNorm algorithm, βactin, B2M, and HPRT were determined to be the best performing housekeeping genes (HKG) and the geometric mean quantities of the HKG were used as the normalization factor [11]. The PCR reactions were performed in 25-μl reaction mixture volumes containing SYBR Green PCR Master Mix (Applied Biosystems), primer pairs, and cDNA. The programs for RT-qPCR were set to 15 min at 95°C, followed by 40 cycles of 17 s at 95°C, 30 s at 54°C, and 30 s at 72°C. Gene expression was calculated using the DDCt method. Following each PCR, dissociation curve analysis was used to evaluate the specificity of product amplification. Primer sequences are listed in (Table A in S1 File). The gene list is given below in Table 1.

**Table 1:** Atherosclerosis Related Gene List (43 genes) for PCR Array.

**Vasoactivity**
eNOS, EDN1(ET-1), PTGS2(COX-2)
**Tight Junctions and Adhesion Molecules**
***Tight junction***: Occludin-1 (OCLN), zonula occludens 1 (ZO-1), VE-cadherin (CDH5)
***Cell-cell adhesion***: ICAM1, SELE (E-Selectin), VCAM1
**Blood Coagulation**
THBD (Thrombomodulin), endothelial protein C receptor (EPCR), CD36, syndecan-1(SCD-1)
**Lipid Metabolism**
ABCA1 (ABC-1), APOE, OLR1 (oxLDL or LOX-1), ADFP (adipophilin), SCARB1 (SR BI)
**Nuclear Receptors and Inflammatory**
***Nuclear receptor***: NR1H3 (LXRA), PPARG
***Inflammatory response***: CCL2, CCL5 (Rantes), NFKB1, IL6, IL8, CD40 (TNFRSF5)
**Oxidative stress**
SOD1, SOD2.
**Apoptosis**
BAX, BCL2, TNFAIP3 (A20), TNFRSF6 (Fas)
**Other Genes**
ANGPT-2, PRDX2, KLF2, BMP4, GPC1, TGFB1, VEGF
**Housekeeping Genes**
GAPDH, 18S, HPRT, B2M, βactin

### Fluorescence inmunostaining

At the end of the experiments silicone membranes with confluent monolayers were stained with specific antibodies for NFKB p50/p105 (Santacruz, California), NFKB p65 (Cell Signaling, Massachusetts) and CDH5 and visualized using fluorescence microscopy. Silicone membranes were washed twice with PBS and fixed in 1% PFA and 4% PFA for NFKB p65 for 10 min, cut into sections, and permeabilized with Triton X-100 in PBS for 10 min. After permeabilization samples were blocked in 10% BSA and 0.1% Triton X-100 in PBS (NFKB and CDH5) for 1h. After washing with 0.1% Triton X-100 in PBS, the silicone sheets were incubated with polyclonal rabbit anti- NFKB, and CDH5 primary antibodies overnight at 4°C followed by washing with 0.1% Triton X-100 in PBS. Samples were subjected to secondary antibody Alexa Fluor 488 donkey anti-rabbit (1:500 to 1:200; Invitrogen) secondary antibody for 1.5 h for CDH5 and with secondary antibody Alexa Fluor 488 donkey anti-goat (1:100 to 400; Santa Cruz) for 1.5h for NFKB. Samples were washed again with 0.1% Triton X-100 in PBS and mounted with vectashield mounting media with DAPI on glass slides with cover slips in contact with cells. These slides were imaged using a Nikon Eclipse TE2000-E inverted fluorescence microscope with a Photometrics Cascade 650 camera (Roper Scientific) and MetaVue 6.2r2 imaging software (Universal Imaging).

### Western blot analysis

The monolayers were washed once with ice-cold PBS and scraped from the silicone membranes with a plastic scraper in the presence of RIPA extraction buffer (1 mM NaHCO3, 2 mM PMSF, 1 mM Na3VO4, 5 mM EDTA, 10% protease and phosphatase inhibitor cocktail tablet and 1% Triton-X) followed by 30 s sonication on ice. Protein concentration was determined with the Protein determination kit from Cayman chemical (Ann Harbor, Mi) using the spectrophotometer Synergy HT from Biotek. Western blotting was carried out by standard techniques, loading 30 μg of protein into gradient precast-gels from Biorad, (Berkeley, Ca) and incubating overnight with antibodies to NFκB (p50/P105 dilution 1:800), NFKB (p65 dilution 1:1000) and CDH5 and the constitutively expressed protein β-actin from Cell Signalling Technologies (Beverly, MA), followed by specific secondary HRP conjugated anti-rabbit, anti-mouse and anti-goat IgG from Cell Signalling Technologies (Beverly, MA). The blots were scanned with the Biorad western blot scanner and quantified with Image J software.

### Hemodynamic Simulator

The hemodynamic simulator is described in detail in (S1 File). Briefly, it is based on the principle of a parallel plate flow chamber [12], but the upper plate is comprised of an elastic silicone membrane with the plated EC that is stretched in a direction perpendicular to the flow direction. A rocker arm mechanism provides the periodic stretching of the upper membrane with a defined strain (S1 & S2 Figs). This mechanism is mechanically linked to a valve that converts a steady flow pressure head into a sinusoidal flow (WSS) with defined mean and amplitude. The phase angle between the strain (CS) and the flow (in phase with the WSS) defines the SPA, as shown in (S2 & S3 Figs). This can be varied by rotating the mechanical linkage between the valve and the rocker arm.

### Plan of experiments

To explore how endothelial cells acquire different phenotypes in response to vascular pulsatile WSS and CS at SPA characteristic of athero-protective and athero-prone regions, we used the hemodynamic simulator to reproduce the same sinusoidal WSS and CS waveforms, but at different phase angles. Changes in the expression of 39 genes, after 7 hours of mechanical stimulation, were studied. This time point was selected for comparison because EC are considered nearly flow-adapted with respect to gene expression after this time exposure. Details of the imposed WSS and CS conditions are given below.

#### Oscillatory flow with highly asynchronous SPA

Oscillatory flow with 10±10 (mean ± amplitude) dyn/cm^2^ WSS, 4 ± 4% CS, frequency = 1 Hz, and SPA of -180° (atheroprone).

#### Oscillatory flow with synchronous SPA

Oscillatory flow with 10±10 dyn/cm^2^ WSS, 4 ± 4% CS, frequency = 1 Hz, and SPA = 0° (atheroprotective).

#### Static control

No hemodynamic forces were applied on the silicon substrate. No flow (WSS = 0 dyn/cm^2^), and no strain (CS = 0%).

Note that in each dynamic case the mean WSS is typical of arterial flow in a non-separated flow zone and that there is no flow reversal (OSI = 0). The CS is in a normal physiological range. The mean and amplitude of WSS and CSS do not vary between conditions, only the SPA.

### Data analysis

Results are presented as mean ± SEM obtained from at least eleven independent experiments for gene expression and independent experiments for Western blots (n = 6 for NFκB, and n = 3 for CDH5). Samples were obtained from monolayers that did not show damage or desquamation. Statistical analysis was performed by one-way analysis of variance (ANOVA) with either the least significant difference (LSD) test or Tamhane’s T^2^ test (depending on Levene’s statistic for homogeneity of variance) using SPSS 20.0 software tool. Difference in means were considered significant if P<0.1.

## Results

### Cell viability and morphology

**Fig 2:**
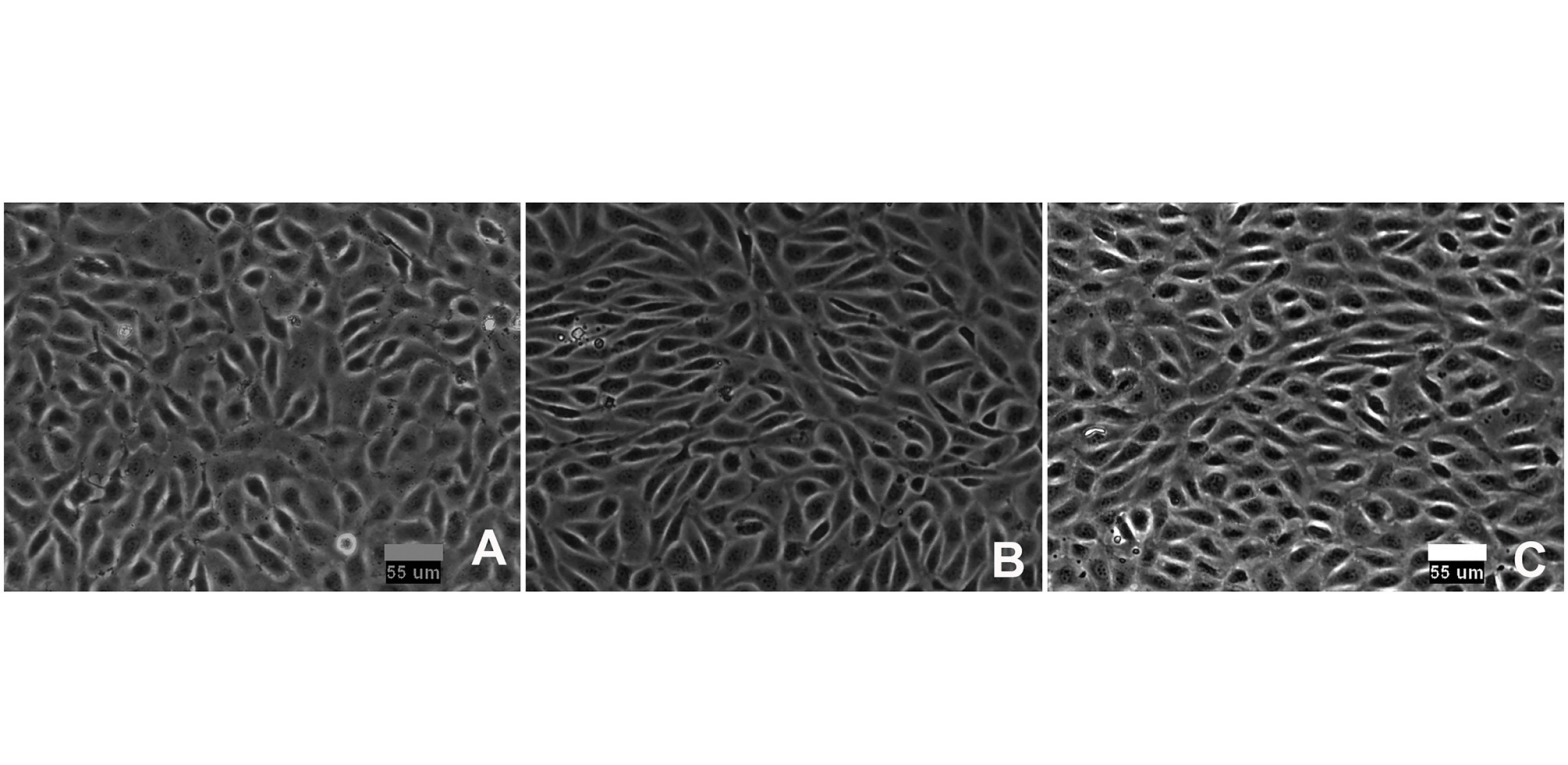
Endothelial cells seeded on the inner surface of silicone substrates. (A) Control conditions. ECs formed a confluent monolayer that has a cobblestone appearance. (B) After 7-h-exposure to WSS (10±10 dynes/cm2) and CS (4 ± 4%) at SPA = 0° and (C) SPA = -180°. ECs remained confluent but showed subtle morphological changes. Flow is from left to right; strain is perpendicular to flow.

BAECs were exposed to WSS (10±10 dyn/cm^2^) and CS (4 ± 4) for 7 h in the hemodynamic simulator. The cells remained viable, with no signs of injury or desquamation, throughout the experiments at SPA = -180° and SPA = 0°. Staining with PI showed no increase in dead cells after exposure to shear stress and stretch (Data not shown). The cells showed morphological changes in response to WSS and CS, becoming partially elongated and aligned parallel to the direction of flow and perpendicular to the CS as shown in Fig 2. These results indicate that the device can be used to apply WSS and CS to endothelial cells without causing cell damage.

### Vasoactivity gene expression

**Fig 3:**
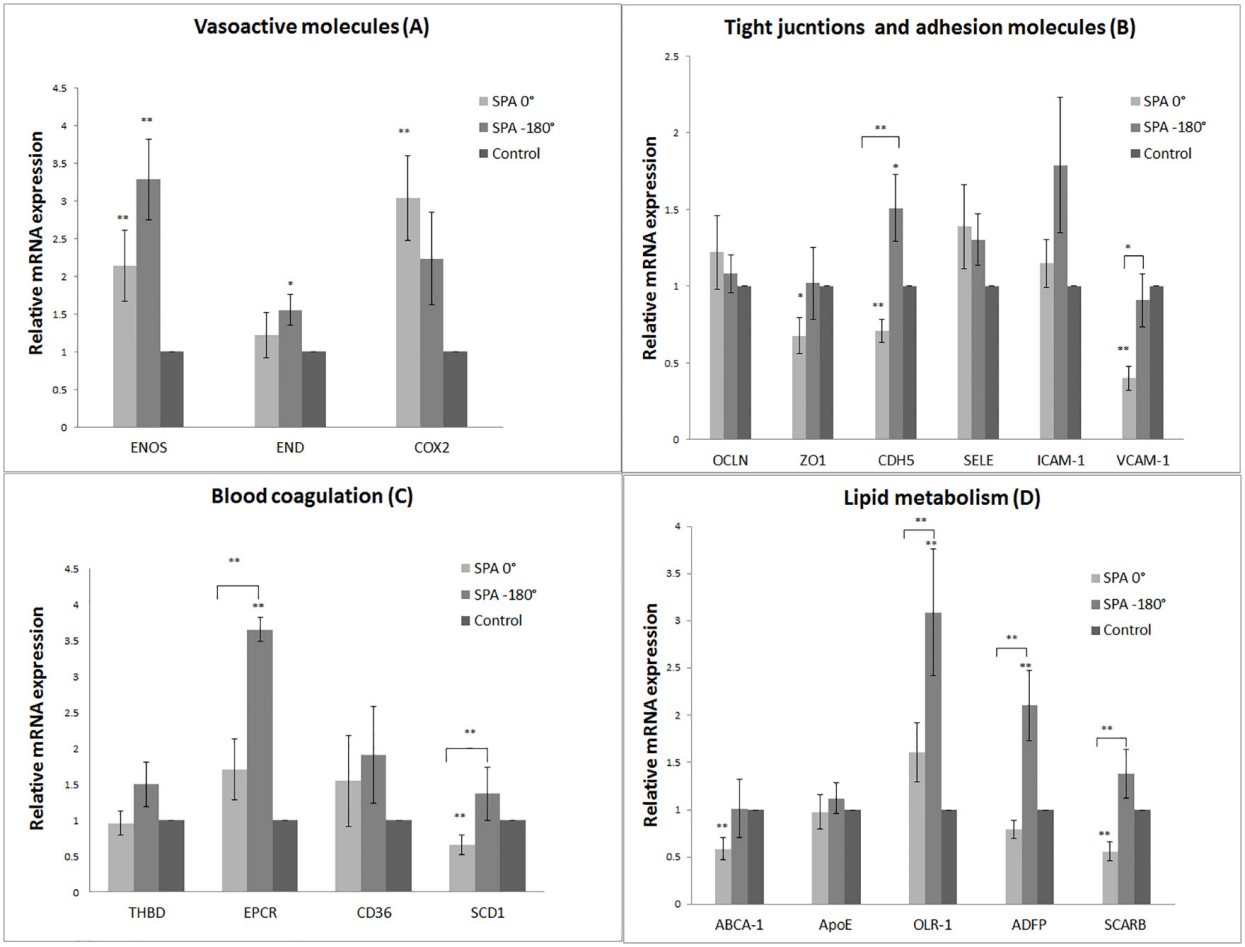
Hemodynamic influences on gene expression. Asynchronous hemodynamic conditions significantly increased the expression of the adhesion molecules CDH5 and VCAM-1 (B). Asynchronous hemodynamic conditions significantly increased EPCR and SCD1 mRNA levels (C). Asynchronous hemodynamic conditions significantly increased the mRNA levels of transcription regulators OLR-1, ADFP, and SCARB1 (D) **p < 0.05, * p<0.1 indicate significant differences for SPA 0° with respect to control or SPA -180° with respect to control. An overbar indicates pairwise significant difference between SPA 0° and SPA -180°. (n = 11)

We analyzed the regulation of mRNA levels for vasoactive genes endothelial nitric oxide synthase (eNOS), endothelin-1 (ET-1) and cyclooxygenase-2 (COX-2). WSS and CS induced 2.3 and 3.3 fold increase in eNOS mRNA levels for SPA = 0° and SPA = -180°, respectively, compared to static control, indicating that BAECs were responsive to WSS and CS (Fig 3A). Asynchronous hemodynamics increased ET-1 mRNA levels ~1.55-fold relative to static controls. Synchronous hemodynamics increased COX-2 mRNA levels ~3.55-fold relative to static control. There were no significant differences between synchronous and asynchronous conditions for any of these genes. WSS and CS showed a significant difference from static controls in ET-1 mRNA levels for SPA = -180° but not SPA = 0°. WSS and CS induced 3.3 and 2.2-fold increases in COX-2 mRNA levels for SPA = 0° and SPA = -180° respectively. Only SPA = 0° induced a significant increase in COX-2 mRNA levels compared with static controls.

### Tight junction and adhesion proteins gene expression

We analyzed tight junction proteins OCLN and ZO-1 and adhesion molecules SELE, CDH-5, ICAM-1, VCAM-1 (Fig 3B). BAECs exposed to asynchronous or synchronous hemodynamic conditions did not show any significant difference in OCLN and SELE mRNA levels. The asynchronous condition appeared to increase ICAM-1 mRNA levels compared with SPA = 0°, but this effect was not significant. Asynchronous hemodynamics significantly increased CDH-5 (2.1-fold) compared with synchronous hemodynamics. Synchronous hemodynamics reduced CDH-5 (~1.4-fold) and ZO1 (~1.5-fold) mRNA levels relative to static controls. Synchronous WSS and CS significantly decreased VCAM-1 (~2.2-fold) mRNA levels compared with asynchronous hemodynamics and static controls. For the asynchronous hemodynamic condition, VCAM-1 mRNA expression stayed at the control level.

### Blood coagulation gene expression

We analyzed the regulation of mRNA levels for the blood coagulation factors Thrombomodulin (THBD/TM), endothelial protein c-receptor (EPCR) and syndecan-1 (SCD1), and the cell surface receptor cluster of differentiation (CD36) as shown in Fig 3C. Asynchronous hemodynamics significantly increased EPCR mRNA levels compared to static controls (3.6-fold). In addition asynchronous hemodynamics increased EPCR mRNA levels compared to the synchronous case (2.1-fold). Synchronous hemodynamics significantly decreased the SDC-1 mRNA levels compared with asynchronous hemodynamics (2.2-fold).

### Lipid metabolism genes expression

We analyzed the regulation of mRNA levels for the lipid transporter ABCA-1, Apolipoprotein E (apoE), oxidized LDL receptor-1 (ORL1), Adipose Differentiation-Related Protein / Adipophilin (ADFP), and Scavenger receptor class B type 1 (SCARB-1 / SR-B1) as shown in Fig 3D. Synchronous hemodynamic conditions significantly reduced ABCA-1 mRNA levels (1.6-fold) compared with control. Asynchronous conditions very significantly augmented ORL-1 mRNA levels by 3.1-fold compared with static controls and by 2-fold compared with synchronous hemodynamics. Asynchronous conditions significantly increased ADFP mRNA levels by 2.8-fold compared with synchronous hemodynamics and by 2.3-fold compared with static controls. Synchronous hemodynamic conditions significantly reduced SCARB-1 mRNA levels (2.5-fold) compared with asynchronous hemodynamics.

### Apoptosis gene expression

**Fig 4:**
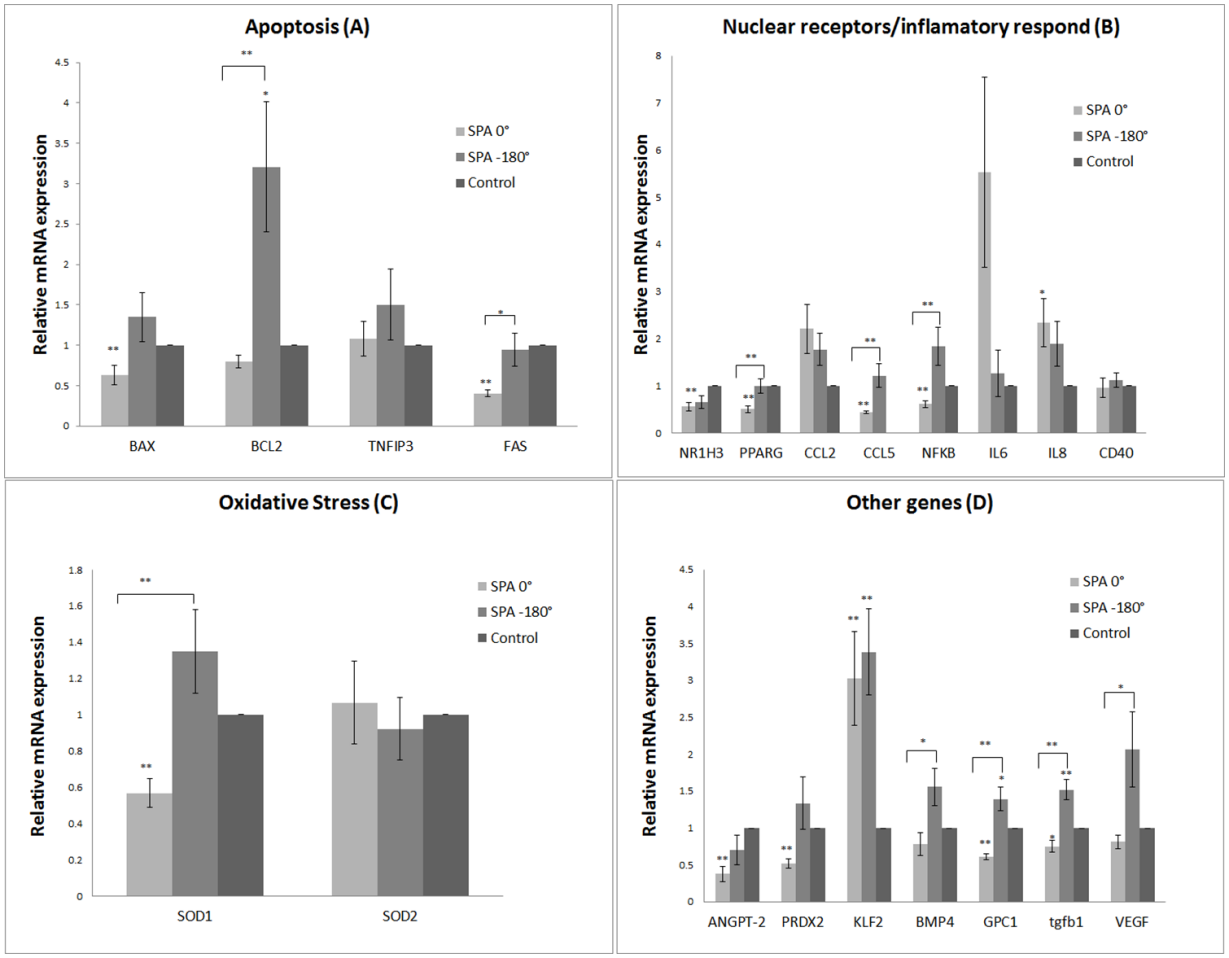
Hemodynamic influences on gene expression. Asynchronous hemodynamic conditions significantly increased the levels of mRNA of the apoptosis factor BCL2. Synchronous hemodynamics significantly reduced BAX and FAS mRNA levels (A). Asynchronous hemodynamics significantly increased the mRNA levels of PPARG, CCL5 and NFκB relative to synchronous conditions (B). Synchronous hemodynamics significantly decreased the mRNA levels of SOD1 compared to controls and SPA = −180° (C). Asynchronous hemodynamics significantly increased the mRNA levels of BMP4, GPC1, TGFb1 and VEGF compared to SPA 0°. Synchronous hemodynamic conditions decreased the gene expression of ANGTP2 and PRDX2 compared to control (D). (n = 11).

We analyzed the gene expression of apoptosis regulating proteins: the BCL2-associated X (BAX), B-cell lymphoma 2 (BCL2), Tumor Necrosis Factor-Alpha-Induced Protein-3 (TNFAIP-3 / A20), and Apoptosis Stimulating Factor (Fas) as shown in Fig 4A. Synchronous condition decreased BAX (~1.7-fold) mRNA levels compared with control. Asynchronous conditions very significantly increased BCL2 mRNA levels by 3.2-fold compared with static control and by 4.0-fold compared to synchronous hemodynamic conditions. Synchronous conditions significantly reduced FAS mRNA levels (~2.5-fold) compared with static control and asynchronous conditions.

### Nuclear receptors and inflammatory response gene expression

We analyzed the regulation of Nuclear Receptor Sub-Family 1, Group H, Member 3 (NR1H3), Peroxisome proliferator-activated receptor G (PPARG) and the inflammatory response genes Chemokine (C-C Motif) Ligand-2 (CCL-2), Chemokine (C-C Motif) Ligand-5 / RANTES (CCL-5), interleukins (IL6, IL8), and cluster of differentiation CD40, and the transcriptional factor Nuclear factor-kappa B (NFκB-1/ NF-κB) as shown in Fig 4B. Synchronous hemodynamic conditions significantly reduced NR1H3 mRNA levels (~1.8-fold) compared with static control. Synchronous conditions significantly reduced PPARG mRNA levels by 2-fold compared with static control and asynchronous conditions. Synchronous hemodynamics significantly reduced CCL5 mRNA levels by 2.3-fold compared with static controls and by 2.8-fold compared to asynchronous hemodynamic conditions. Synchronous conditions significantly reduced NFκB mRNA levels by 1.62-fold compared with static controls and by 3-fold compared to asynchronous conditions. Synchronous conditions increased IL-8 mRNA levels by 2.34-fold compared with static controls.

### Oxidative stress gene expression

We analyzed the regulation of Superoxide Dismutase-1 / Cu,Zn Superoxide Dismutase (SOD-1) and Superoxide dismutase-2 (SOD2) (Fig 4C). Synchronous conditions significantly reduced SOD1 mRNA levels by 1.8-fold compared with static control and by 2.3-fold compared to asynchronous conditions. There were no significant effects on SOD2.

### Other genes

We analyzed the regulation of Angiopoietin-2 (ANGPT-2 /Ang-2), peroxiredoxin-2 (PRDX-2), Kruppel-like factors 2 (KLF2), bone morphogenetic protein-4 (BMP4), glypican-1 (GPC-1), transforming growth factor beta-1 (TGFb1) and vascular endothelial growth factor (VEGF) (Fig 4D). Synchronous conditions significantly decreased ANGPT-2 mRNA levels by 2.6-fold compared with static controls. Synchronous conditions significantly reduced PRDX-2 mRNA levels (~2-fold) compared with static controls. Asynchronous and synchronous conditions significantly increased KLF-2 mRNA levels by 3.0-fold and 3.4-fold, respectively, compared with static controls, but they were not different from each other. Asynchronous conditions significantly increased BMP4 mRNA levels by 2.0-fold compared with synchronous conditions. Asynchronous conditions significantly increased GPC-1 mRNA levels by 1.4-fold compared with static control and by 2.2-fold compared to synchronous conditions. Asynchronous conditions significantly increased TGFb1 mRNA levels by 1.5-fold compared with static control and by 2.0-fold compared to synchronous hemodynamic conditions. Asynchronous conditions significantly increased VEGF mRNA levels by 2.5-fold compared to synchronous hemodynamic conditions.

### NFκB staining in BAECs exposed to SPA = 0° and SPA = -180°

**Fig 5:**
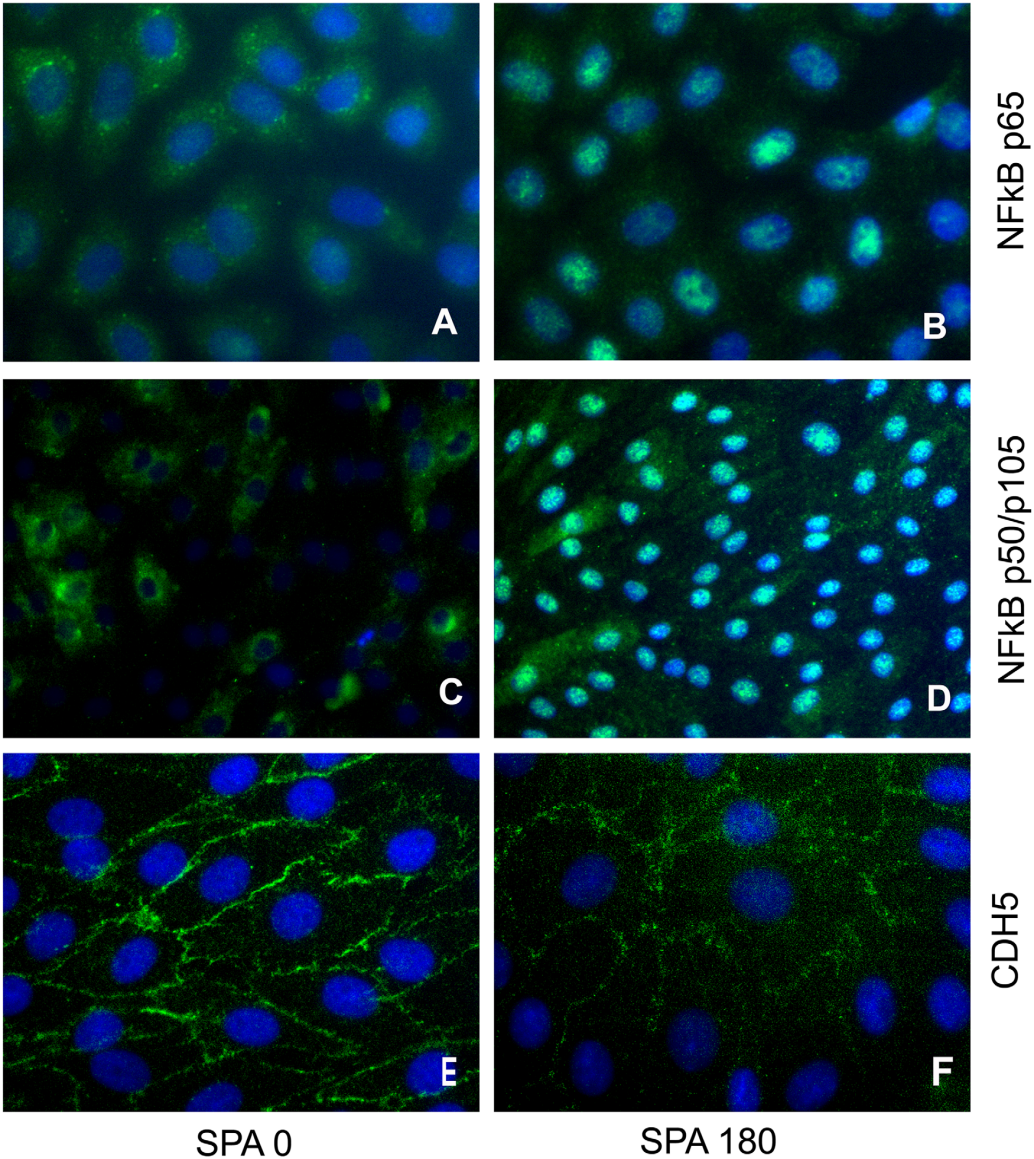
SPA modulates localization of NFKB p105/p50, NFKB p65 and CDH5. BAEC were exposed to asynchronous or synchornous condition for 7h. Stainings for NFKB p105/p50 (B) and p65 (D) were localized in the citoplasm and the nucleos for EC exposed to SPA = -180. NFKB localization where entirely citoplasmatic for EC exposed to SPA = 0 (A, C). The distribution for CDH5 were continous around the entire periphery of the cells after 7 when cells where exposed to SPA = 0 (E). Exposure of EC to SPA = -180 for 7 hours resulted in an intermitted pattern of CDH5 (F). images showed here are representative resutl from 3 individual experiments.

NFκB gene expression was significantly up-regulated by SPA = −180° compared to SPA = 0° (Fig 4B). To assess the effects of SPA on the protein expression and localization of NFκB p105/p50 and NFKB p65 we compared the immunostaining of NFκB (p105/p50 and p65) on BAECs exposed to asynchronous or synchronous hemodynamics for 7h. Our results indicate that asynchronous hemodynamics induce the translocation of NFΚB p105/p50 and p65 to the nucleus as shown in the Fig 5b and 5e. The localization of NFκB is entirely cytoplasmic for synchronous hemodynamics (Fig 5a and 5d).

### CDH-5 staining in BAECs exposed to SPA = 0 and SPA = -180 in vitro

CDH5 (VE-cadherin) gene expression was significantly up-regulated by SPA = −180 compared to SPA = 0 (Fig 3B). To assess the effects of SPA on the protein expression and localization of CDH5, we compared the immunostaining of CDH5 on BAECs exposed to asynchronous and synchronous hemodynamics. Under synchronous conditions for 7h, CDH5 staining was continuous and distributed around the entire periphery of endothelial cells (Fig 5F) indicating robust adherens junction formation. In contrast, after exposure to asynchronous hemodynamics for 7h, CDH5 staining at cell-cell junctions became intermittent and in some areas not detectable (Fig 5G) indicating weakened adherens junctions.

### Relative Protein expression of NFKB and CDH5

**Fig 6:**
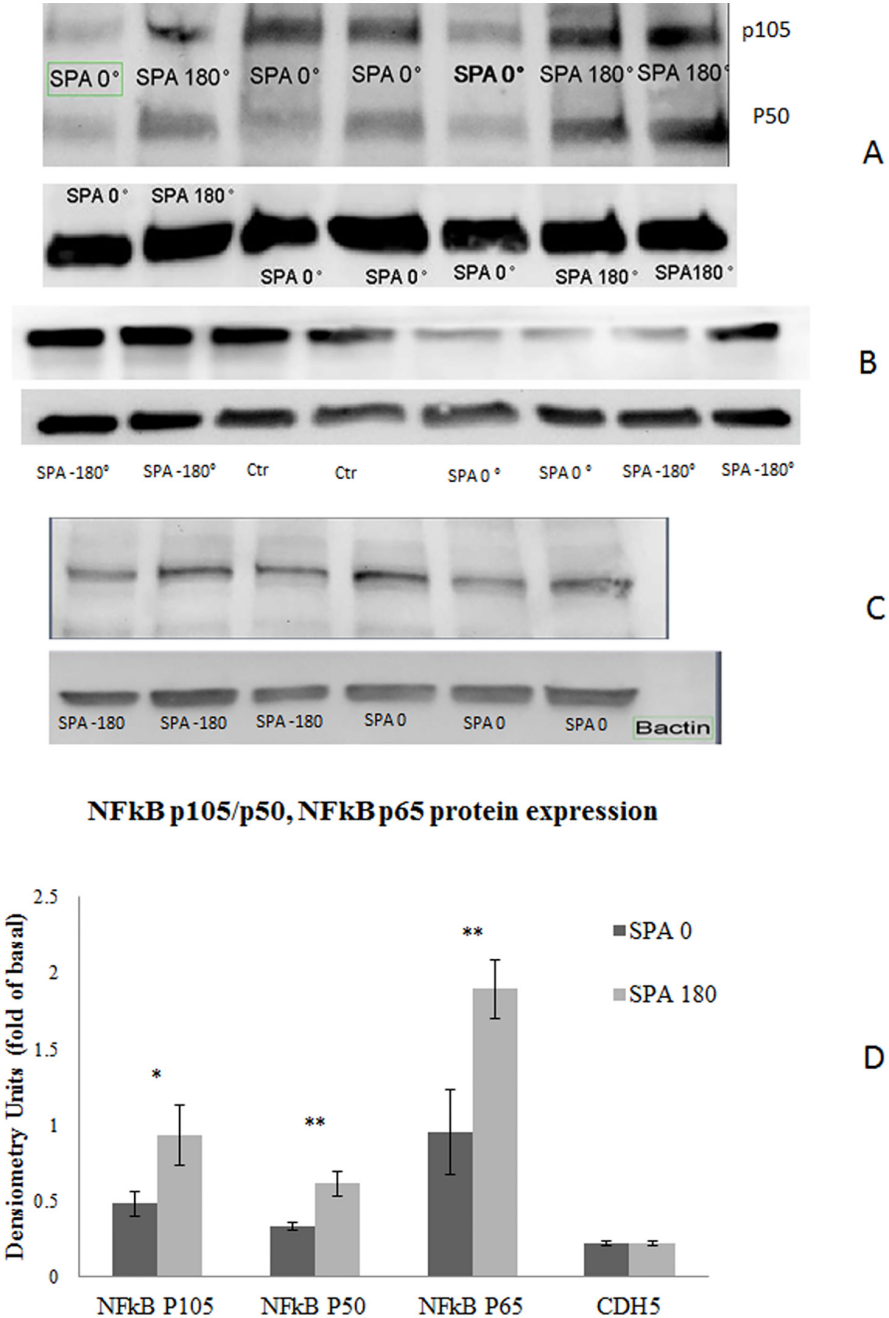
SPA modulates the protein expression levels of NFkB p105/p50 and NFkB p65 but does not affect CDH5. BAEC monolayer were exposed to WSS and CS with either SPA = 0 or SPA = -180 during 7 hours. Cell lysates from different samples (n = 6 each condition) were separated in gradient SDS-PAGE, and the proteins were transferred to nitrocellulose membranes. Nitrocellulose membranes were split into two parts for immunoblotting with NFKB p105/p50 or NFkB p65 using βactin as the endogenous control. Representative blots are shown in Fig 6. Samples were analysed by densitometry and normalized by the βactin control; then the relative protein expressions at SPA = -180 and SPA = 0 were compared. The bar graphs in (A) represent the quantification of 6 individual experiments (mean ± SEM). SPA = -180 increases the expression of NFκB p50 by 1.9 fold compared to SPA = 0 (p = 0.001) and the expression of NFκB p105 by 1.98 fold (p = 0.058). The bar in (B) suggest that SPA = -180 increase the expression of the transcriptional factor NFKB p65 (n = 8) by 1.98 fold (p = 0.002) (ANOVA, all p<0.05). SPA did not affect the CDH5 protein expression levels (n = 3 for each condition) (ANOVA p>0.05) as shown in panel B.

Asynchronous conditions increased the level of protein expression for NFκB p105, p50 and p65 compared to synchronous hemodynamics (Fig 6). These observations are consistent with the hypothesis that asynchronous hemodynamics is atheroprone and indirectly indicate EC under asynchronous hemodynamics initiate an inflammatory state. We found that asynchronous conditions significantly increased protein expression of NFκB p50 by 1.9 fold (p = 0.001) and NFκB p105 by 1.98 fold (p = 0.058) compared to SPA = 0° similarly, asynchronous hemodynamics increased the protein expression of NFκB p65 by 1.98 fold (p = 0.02) compared to SPA = 0°. SPA = 0° or SPA = -180° did not have any effect on the level of protein expression of CDH5 (p = 0.3).

## Discussion

Most in vitro studies of the role of hemodynamics in atherosclerosis have emphasized the analysis of isolated forces, mainly fluid shear stress, but also cyclic stress (strain). Different hemodynamic conditions such as low mean wall shear stress, disturbed flow, and reversal flow (high OSI) have been associated with the localization and development of atherosclerosis. The coronary arteries, the locations most prone to atherosclerosis, exhibit highly asynchronous hemodynamic conditions (SPA close to -180°); approximately 50% of the total coronary blood flow occurs in early diastole, 25% in late diastole, and only 25% in systole [13]. SPA close to -180° also occurs on the outer wall of bifurcations and other characteristic atheroprone sites due to geometric factors [3]. Hypertension, vessel stiffness due to aging, and the increase in the severity of stenosis are major risk factors in several cardiovascular diseases. These factors have been shown to induce a more negative SPA [14, 15].

Asynchronous and synchronous hemodynamic waveforms differentially regulate endothelial gene expression. We have shown that identical WSS and CS waveforms elicit different gene expression profiles depending only on differences in the stress phase angle. Statistical analysis based on duplicate sets of cDNA template experiments using eleven different biological samples from each experimental condition (SPA = 0° and SPA = -180) revealed that 17 out of 38 genes were up-regulated by asynchronous hemodynamics relative to synchronous hemodynamics and classified as atheroprone, atheroprotective or of undetermined role in the disease (Table 2). Notably, no genes were upregulated significantly by synchronous conditions relative to asynchronous hemodynamics.

**Table 2:** Endothelial genes up-regulated by the athero-prone condition SPA = -180° relative to the athero-protective condition SPA = 0°.

Gene Bank N°	Gene name	Atheroprone/atheroprotective ratio	Characteristic	p-Value
BC151459.1	Vascular Cell Adhesion Molecule-1 (VCAM-1)	2.3	Proatherogenic	0.073
M32976	Vascular Endothelial Growth Factor (VEGF)	2.5	Proatherogenic	0.099
XM_002694894	Cadherin-5 (CDH-5 / VE-Cadherin)	2.1	Proatherogenic	0.013
NM_174132.2	Oxidized Low Density Lipoprotein Receptor-1 (OLR-1)	2.7	Proatherogenic	0.047
BC102211	Adipose Differentiation-Related Protein / Adipophilin (ADFP)	2.8	Proatherogenic	0.01
BC102064	Chemokine (C-C Motif) Ligand-5 / RANTES (CCL-5)	2.8	Proatherogenic	0.034
DQ464067	Nuclear Factor of Kappa(NFKB-1 / NF-kB)	3.0	Proatherogenic	0.04
BC105344	Bone Morphogenetic Protein-4 (BMP-4)	2.0	Proatherogenic	0.066
M_001166068	Transforming growth factor b1 (TGFB1)	2.0	Proatherogenic	0.001
NM_174662	Apoptosis Stimulating Factor (Fas)	2.3	Proatherogenic	0.097
BC134513	Scavenger Receptor, Class B, Type 1 (SCARB-1 / SR-B1)	2.5	Antiatherogenic	0.033
BC102432	Superoxide Dismutase-1 (SOD-1)	2.4	Antiatherogenic	0.023
BC116074	Endothelial Protein C Receptor (EPCR)	2.1	Antiatherogenic	0.013
NM_001075924	Syndecan-1 (SDC-1)	2.5	Indeterminate	0.028
NM_181024	Peroxisome Proliferator-Activated Receptor-g (PPAR-G)	2.0	Indeterminate	0.043
NM_001166486	B-Cell CLL/Lymphoma-2 (BCL-2)	4.0	Indeterminate	0.04
BC105496	Glypican-1 (GPC-1)	2.3	Indeterminate	0.002

Asynchronous hemodynamic conditions elicited an up-regulation of genes across all the different gene groups. Direct comparison of SPA = 0° and SPA = -180° conditions illustrated the differences in gene profile pattern. Most significantly, we showed that asynchronous hemodynamics (SPA = -180°) evoke mostly athero-prone genes relative to synchronous hemodynamics (SPA = 0°).

Asynchronous hemodynamics induced the expression of the transcription factor NFκB p50/p105 and p65 which plays a central role in the regulation of inflammation, adhesion and proliferation genes- all precursors to the onset of atherosclerosis [16–18]. In our study we have shown that asynchronous hemodynamics upregulated the expression of the following possible NFκB-1 target genes: VCAM-1 [19] which plays a major role in the initiation of atherosclerosis by facilitating the recruitment of monocytes to the arterial intima [20]; VEGF [21] which induces the migration and proliferation of monocytes and endothelial cells, vascular permeability and thrombogenicity [22–24]; ORL-1 [25] responsible for binding and uptake of oxLDL in endothelial cells facilitating inflammation and atherosclerotic plaque formation, progression and destabilization [26, 27]; TGFB1 [28] which could accelerate atherosclerosis by increasing vascular extracellular matrix accumulation and lipid retention [29]; CCL-5 [30] involved in the recruitment of inflammatory cells onto the vessel wall and proliferation of SMC in atherosclerotic plaques [31, 32]; BCL-2, an anti-apoptotic mediator of vascular cell apoptosis in response to oxidation and inflammation [33]; BMP4 [34] which activates the inflammatory response and enhances the expression of other known proatherogenic genes [35]; FAS [36] which is a pro-apoptotic gene that accelerates atherosclerotic lesion by increasing cellular proliferation and inflammation [37]. In addition, asynchronous hemodynamics increased the gene expression of athero-prone ADFP which increases lipid accumulation and prevents lipid efflux [38].

In contrast with the above results, asynchronous hemodynamic seems to activate an atheroprotective mechanism as well. Asynchronous hemodynamics increased the mRNA levels of three atheroprotective genes: SCARB-1, which is a cell-surface HDL receptor that mediates HDL cholesterol efflux reducing atherosclerosis progression [39]; SOD-1 is one of three superoxide dismutases that destroys free superoxide radicals and whose overexpression inhibits angiotensin II-induced expression of MCP1 and monocyte infiltration [40]. In addition, asynchronous hemodynamics increased the gene expression of EPCR which is crucial for the control of thrombosis [41] and is down regulated in atherosclerotic arteries, causing inflammatory cell migration into plaque regions and thrombosis and cell adhesion molecule CDH5 which plays a role in new vessel formation and inflammatory cell migration into the intima [42].

Asynchronous hemodynamics also increased the expression of genes having both pro- and anti-atherogenic features (indeterminate): Glycocalyx core proteins SDC-1, which is involved in events of cell migration, proliferation, early inflammatory response and matrix remodeling [43], as well as the clearance of triglycerides [44]; GPC-1, which promotes angiogenesis, cellular growth, differentiation, adhesion, migration, and binds VEGF and fibroblast growth factor (FGF) [45, 46], but is also known to activate eNOS in early stages of atherosclerosis [47]; and PPARG that shows proatherogenic features such as enhancing LDL uptake but primarily limits the progression of atherosclerosis by inhibiting VCAM1 expression and monocyte migration [48]

Athero-prone waveforms alter the endothelial apoptosis state. Asynchronous hemodynamics increases the gene expression of FAS (pro-apoptotic) and BCL-2 (anti-apoptotic). The overall effects of these genes in the development of the disease are undetermined. A previous study showed that asynchronous hemodynamics increases apoptosis rate [49] which has been shown to increase endothelial permeability to LDL [50] leading to progression of disease.

Our results did not show any significant difference in mRNA levels for the tight junction protein genes OCLN-1 and ZO-1. A previous study showed that both synchronous and asynchronous hemodynamics had no effect on either OCLN-1 or ZO-1 protein expression after 12 hours [47].

Our results showed that ET-1 mRNA levels were not significantly altered from the basal level under any hemodynamic condition. These results agree with previous studies where it was observed that ET-1 gene expression was not significantly affected at SPA = 0° or -180° after 5 hours [51]. Another study at unknown SPA (probably synchronous) showed that combined WSS and CS did not influence ET-1 mRNA expression, while isolated WSS decreased the gene expression of ET-1, and CS alone increased its expression [52, 53].

Both asynchronous and synchronous hemodynamics significantly increased the expression of three atheroprotective genes relative to static controls: ENOS, COX2 and KLF2 (Figs 3A and 4D). ENOS and COX2 are vasodilators that play an important role in homeostasis; KLF2 is a transcriptional factor which is more abundant in the high-shear regions resistant to atherosclerosis than in areas of disturbed flow and endothelial dysfunction. KLF2 inhibits the proinflammatory response of monocytes and lipid uptake, and inhibits NFκB transcriptional activity [54]

We also examined the expression and distribution of proteins whose genes were up-regulated by asynchronous relative to asynchronous conditions (Figs 5 and 6). In regard to CDH5, in atheroprone regions such as major curvatures and bifurcations the organization of intercellular junctions is not well defined resulting in increased EC layer permeability and vulnerability to leukocyte transmigration. In vivo studies of immunostaining of CDH5 have shown that it is highly expressed at EC borders in the abdominal aorta [55], which is a region protected from atherosclerosis with more synchronous hemodynamics (SPA = −45°) [56], but CDH5 staining is markedly reduced in the stenotic region of arteries with stenosis and not detectable downstream of the stenotic region [55]. These regions are characterized by highly negative of SPA reaching values of SPA = -180° downstream of the stenosis [57]. In the present study we observed that asynchronous hemodynamic upregulated the gene expression of CDH5 (Fig 3B), however this did not translate into upregulation of protein expression (Fig 6). But immunofluorescence staining (Fig 5E and 5F) showed a dramatic change in the localization of CDH5 and junctional reorganization that is similar to that observed in atheroprone regions. These are post-translational modifications that would not be indicated by gene expression. Our results suggest that CDH5 localization and organization may be regulated by the SPA.

On the other hand we found that SPA = -180° increased the protein expression level for NFκB p105/p50 and NFkB p65 compared to SPA = 0° (Fig 6), and this was consistent with the upregulation of gene expression for NFκB (Fig 4B). Interestingly, our results also indicated that NFκB p105/p50 and NFκB p65 are translocated to the nucleus when EC are under asynchronous hemodynamics while it is totally localized in the cytoplasm when EC are under synchronous hemodynamics (Fig 5A and 5B). It is well known that NFκB resides in the cytoplasm of the cell and upon activation translocates to the nucleus [58, 59]. Thus we have shown that asynchronous SPA induces a similar atherogenic translocation of NFκB.

In conclusion, we have reported the results of novel experiments in which BAECs were exposed to a well-defined combination of WSS and CS, accurately simulating synchronous (athero-protective), and asynchronous (athero-prone) hemodynamics. We have shown that asynchronous hemodynamics up-regulate many atheroprone genes including those that modulate pro-inflammatory signal transduction or produce components that enhance the binding of lipoproteins. Of special note, we showed that endothelial cells exposed to asynchronous hemodynamics acquire a pro-inflammatory phenotype with enhanced expression of the important chemokine CCL5 and the activation of NFκB as well as the activation of adhesion molecules such as VCAM-1 and several growth factors including VEGF and TGFβ1. We also reported post translational modifications of CDH5 and NFκB induced by asynchronous SPA that are consistent with an atheroprone environment. Based on these results, the SPA appears to be an important parameter characterizing the hemodynamic environment.

## Supporting Information

S1 File Hemodynamic simulator and strain and flow characterization. Analysis of the flow dynamics in the novel device and strain characterization of silicone substrate sheets. Table A in S1 File. PCR primer sequences.
(DOCX)

S1 Fig Schematic of the hemodynamic simulator test section and the valve phase link station. The bioreactor combines a customized pulsatile flow valve mechanically linked to a membrane stretching mechanism and a parallel flow chamber. Different SPA values can be generated by changing the configuration of the phase link station.
(TIF)

S2 Fig Strain characterization. Displacement over time of reference markings recorded by video, showing a sinusoidal displacement when the reference markings were tracked over 8 complete cycles for a CS = 4 ± 4%. The strain characterization of the silicone substrate using computational software ABAQUS determined a uniform strain distribution in the center of the flow channel at a maximum strain of 10%. The cells were plated in the uniform strain region.
(TIF)

S3 Fig Fluid flow analysis in real-time by data acquisition using software written in LabVIEW. The upper panel of the LabVIEW screen shows a typical flow waveform and the lower panel shows the associated FFT indicating very little contribution from the second or higher harmonics.
(TIF)
