# Supplementary material for: The Interaction between Fluid Wall Shear Stress and Solid Circumferential Strain Affects Endothelial Gene Expression
Source: PLoS One. 2015 Jul 6;10(7):e0129952. doi: 10.1371/journal.pone.0129952 (PMC4492743; doi:10.1371/journal.pone.0129952)
Supplement: S1 File — Analysis of the flow dynamics in the novel device and strain characterization of silicone substrate sheets. Table A in S1 File. PCR primer sequences. (DOCX) [file pone.0129952.s001.docx]

# Supporting material and methods

## Hemodynamic Simulator

To investigate the role of the SPA parameter in hemodynamics, it was necessary to develop a new device capable of controlling pulsatile wall shear stress (WSS) and cyclic strain (CS) over a wide range of physiological conditions. The novel device combines a customized pulsatile flow valve that is mechanically linked to the membrane stretching mechanism, and a parallel-flow chamber (S1 Fig). The flow is driven by hydrostatic pressure into the pulsatile valve that has been designed to impose an oscillatory (nearly sinusoidal) component on the steady flow that results in a range of pulsatile conditions with the capability of controlling WSS mean, amplitude and frequency. The fluid flow waveform is driven into a parallel-flow chamber that is comprised of a flow channel, where the upper wall is a moving flexible substrate where EC are seeded on a delimited region facing the pulsatile flow media. The flexible substrate is set totally flat by a central clamping holder (neighboring the parallel-plate flow chamber) and a stretching actuator to avoid internal leaks. The silicone substrate is submerged in experimental media during the experiment, which results in a negligible frictional force when it is in motion

The mechanical motion in the device is driven by a DC motor and the mechanical forces are transmitted simultaneously to both the pulsatile valve and the stretching actuator through a mechanical linkage. A phase angle-link station that connects the motor with the pulsatile valve allows different configurations in the opening of the valve, generating any desired phase angle between the pulsatile flow and the stretching actuator (S1 Fig). This is how the SPA is controlled while WSS and CS waveforms are maintained constant. The novel device is capable of applying different rates of sinusoidal WSS with mean WSS and WSS amplitude in the range (0 – 13 dyn/cm^2^ ) and the tensile cyclic strain in the range 0% - 20%. The frequency range is (0 to 2 Hz).

## Strain and Flow Characterization

Characterization of the strain in the silicone substrate was determined by making a video recording of a grid of reference markings placed on the silicone substrate. A camera recorded the displacement of these reference markings during several cycles, and the values obtained were used to calculate the strain based on an initial reference distance. A graph of the displacement of two neighboring points near the center of the membrane shows that strain values are sinusoidal with nearly constant amplitude cycle by cycle (S2 Fig). A strain characterization study using finite element analysis software ABAQUS determined the most accurate area for cell culture (uniform strain) as shown in S2 Fig.

The Real-time monitoring of flow was accomplished using a flow meter and a non-invasive ultrasound probe (Transonic Ts410). Sensor signals were acquired in real time with custom data acquisition software written in Labview using a DAQ card (200kHZ, 6036E). The software analyzes the WSS waveform using FFT analysis and displays frequency, peak and mean values of WSS. The measured flow (Q) is used to calculate the WSS using the parallel plate flow chamber formula WSS = 6µQ/bd^2^ , where μ is the fluid viscosity, b is the width of the channel and d is the height of the channel. A typical flow waveform and its FFT are shown in S3 Fig. The FFT demonstrates that the waveform is nearly sinusoidal.

## Table A. PCR primer sequences.

| **Vasoactivity** | **Reverse primer (5' to 3')** | **forward primer (5' to 3')** |
| --- | --- | --- |
| Endothelial Nitric Oxide Synthase (eNOS) | AGC AGA CCC CGT AGT GTA G | AGC CCC GGT ACT ACT CT T |
| Endothelin-1 (EDN1 / ET-1) | GCT GGG CTT TCA GCT TGG CA | TCT TCA TCA GCA GCT CGT GGC |
| Cyclooxygenase-2 (COX-2) | TTG TGC CCT GGG GAT CAG GAA | TGC CTG ATG ACT GCC CAA CAC |
| **Tight junction / adhesion** |  |  |
| Occludin (OCLN) | TGT TCC ATA GCC AA ACC GT | GAA CCT TAA TGG GAG CTG GT |
| Zonula Occluden-1 (ZO-1) | ATC ACC CAC AGC GGA TTC TA | GTC CTC TTC CTG CTT GAC CT |
| Cadherin-5 (CDH-5 / VE-Cadherin) | TGC TCA GGG AGA ACT TGA AC | AGC CCA AAG TGT GTG AGA AC |
| E-Selectin (SELE) | TTG AAG GCA AAG TCT CCA AC | AAG CAG CCA ACA TGT AAA GC |
| Intercellular Adhesion Molecule-1 (ICAM-1) | CGT GAG CCT ACT TCC ACA AT | TGG AAC TCT TCC AGA ACA CC |
| Vascular Endothelial Growth Factor (VEGF) | CAC ATC TGC AAG TAC GTT CG | GTT TGT ACA AGA TCC GCA GAC |
| **Blood coagulation** |  |  |
| Thrombomodulin (THBD / TM) | TGA GTT GGT GGA CGG CGA GT | CTC GGC GCA GAT GCA CTT GT |
| Endothelial Protein C Receptor (EPCR) | CCA GGC CGC AAT GTC TCG AT | TGC ACC AAG CCC ACG AAC TC |
| Cluster of Differentiation 36 (CD36) | CAG CGG TGA TTT GAC CCA GCA | GCC ACC AGT GTC AAC GCA CT |
| **Lipid metabolism / transcription regulators** |  |  |
| ATP-Binding Cassette (ABCA-1) | TGG AGC TGG CAA GAC CAC CA | TGG TGC TCA TCT CGG AGC GT |
| Apolipoprotein E (ApoE) | AAG GAC AGC CAG CCT TGG GA | TCC ATC AGC GCC GTC AGT TC |
| Oxidized Low Density Lipoprotein Receptor-1 (OLR-1) | TCA GCA AGT GGG CAT CCA AA | GGT CCT TGT CCC CAA GAC TG |
| Adipose Differentiation-Related Protein (ADFP) | ACC ACC CCC GTC ATT GTG CT | TGG CCA ATG CCA AAG GGG CT |
| Scavenger Receptor, Class B, Type 1 (SCARB-1) | AAG TCT CTC AGC AGC GGC TCC A | TGC ACA TTG CAC GCA CGC AC |
| **Nuclear receptors** |  |  |
| Nuclear Receptor / Liver X Receptor-NR1H3 | GCG CAG GGC AAA CAC TTG CT | ACC ACC CCC ATG ACC GAC TGAT |
| Peroxisome Proliferator-Activated Receptor-G (PPAR-G) | GCA GCT TGG CAA AGA GCT GGG A | AGA CCG CCC AGG TTT GCT GA |
| **Inflammatory response** |  |  |
| Monocyte Chemoattractant Protein-1 (MCP-1) | TAG CAG CAG GCG ACT TGG GAG | AAC ATG AAG GTC TCC GCT GCCC |
| Chemokine (C-C Motif) Ligand-5 / RANTES (CCL-5) | AAA GCA GCA GGG CGT GGT GT | AAG CCT CTG CCC ACA GCT ACCA |
| Nuclear Factor of Kappa(NFΚB-1 / NF-kB) | AAA GAG GGC GTT GGC GTG CT | AGG GGC ACT GCA GCT AAC CTG A |
| Interleukin-6 (IL-6) | AGT CTG CCT GGG GTG GTG TCA T | CCG CTT CAC AAG CGC CTT CA |
| Interleukin-8 (IL-8) | TGG GGT GGA AAG GTG TGG AAT | TGG CTG TTG CTC TCT TGG CAG C |
| Tumor Necrosis Factor (TNF) |  |  |
| Cluster of Differentiation 40 (CD40) | ACA AGC TGT GGG GCG TGC AA | TCC AGA GCG AGG GCA CCT TGA A |
| **Oxidative stress** |  |  |
| Superoxide Dismutase-1 (SOD-1) | ACC ACC ATC GTG CGG CCA AT | AGG CAT GTT GGA FAC CTG GGC A |
| Superoxide Dismutase (SOD-2) | TTC AAC GCA GGC TGC AGA GC | CAG CTG CAC CAC AGC AAG CA |
| **Apoptosis** |  |  |
| B-Cell CLL/Lymphoma-2 (BCL-2) | GCA GGC CTG TGG GCT TCA CTT A | AGC ATG CGG CCC CTG TTT GA |
| BCL-2 Associated X Protein (BAX) | CGG TGA GCA CTC CAG CCA CAA A | TTG GAC TTC CTT CGA GAC CGG C |
| Tumor Necrosis Factor-Alpha (TNFAIP-3 / A20) | AAG TTG CGC GTG TCC GTC TC | ATG CAC GCG GCT TCC CAG TA |
| Apoptosis Stimulating Factor (Fas) | TGG AGA AGG CAA TGG CAC CCC A | TCG CCC AGT CAT GTC CGA CTC A |
| **Other proteins** |  |  |
| Angiopoietin-2 (ANGPT-2 / Ang-2) | TGC ACG GCG GCG TTA GGC ACG TA | ATG GAC ATC GCC GCT GGG AA |
| Peroxiredoxin-2 (PRDX-2 / PRX II) | AGA GCC CAA GCT TGC CAC CA | TGG ACA CCA GGC AGT GAC ACA |
| Kruppel-Like Factor-2 (KLF-2) | TTC ATG TGC AGC GCC AGG TG | TGG AAG TTC GCG CGA TCG GA |
| Bone Morphogenetic Protein-4 (BMP-4) | TGC GTC GGG TCA AGG CAT GT | ACC AGG GCC AGC ATG TCA GGA T |
| Glypican-1 (GPC-1) | AGG GCC TTG GCG TTC TGC GT | AGC AGC GGG GCT TTG ACG A |
| Syndecan 1 (SDC-1) | ATC ACA CAC GCC CTC CAC CT | TGC TCT CCG GTT GGC AGG AT |
| Transforming growth factor ß1 (TGFB1) | AAA ACT TGG CCG CGG ATG GC | TGT GGC TGC TAA TGC TGA CGC C |
| **Housekeeping genes** |  |  |
| HPRT | GCT CGC AAC CTT GAC CAT CT | GAC CAG TCA ACA GGC GAC AT |
| B2M | TCG GCA GCT GTA CTG ATC CT | GGG TTC CAT CCA CCC CAG AT |
| Bactin | CCG TGG TGG TGA AGC TGT AG | CAT GTA CGT GGC CAT CCA GG |
