# Supplementary figures and images for: The Interaction between Fluid Wall Shear Stress and Solid Circumferential Strain Affects Endothelial Gene Expression
Source: PLoS One. 2015 Jul 6;10(7):e0129952. doi: 10.1371/journal.pone.0129952 (PMC4492743; doi:10.1371/journal.pone.0129952)

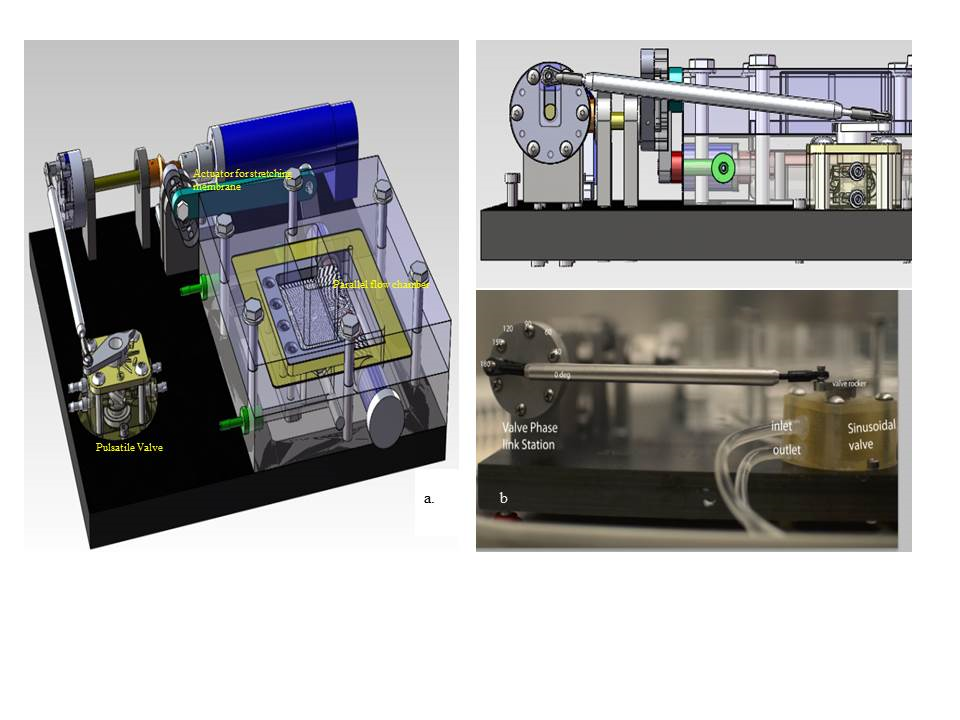

Supplement: S1 Fig — The bioreactor combines a customized pulsatile flow valve mechanically linked to a membrane stretching mechanism and a parallel flow chamber. Different SPA values can be generated by changing the configuration of the phase link station. (TIF) [file pone.0129952.s002.tif]

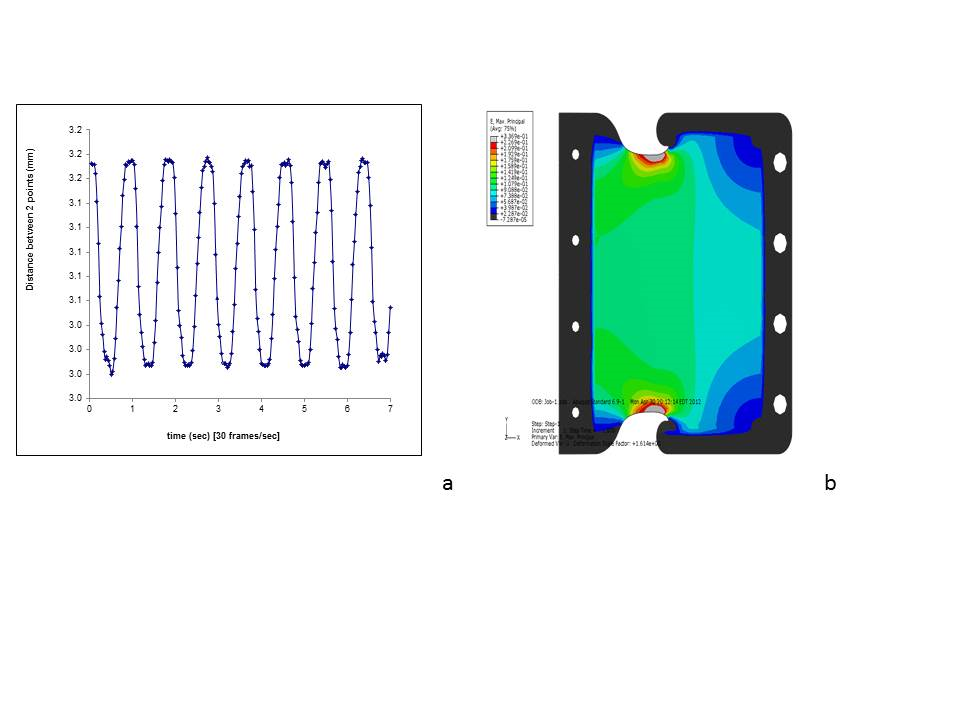

Supplement: S2 Fig — Displacement over time of reference markings recorded by video, showing a sinusoidal displacement when the reference markings were tracked over 8 complete cycles for a CS = 4 ± 4%. The strain characterization of the silicone substrate using computational software ABAQUS determined a uniform strain distribution in the center of the flow channel at a maximum strain of 10%. The cells were plated in the uniform strain region. (TIF) [file pone.0129952.s003.tif]

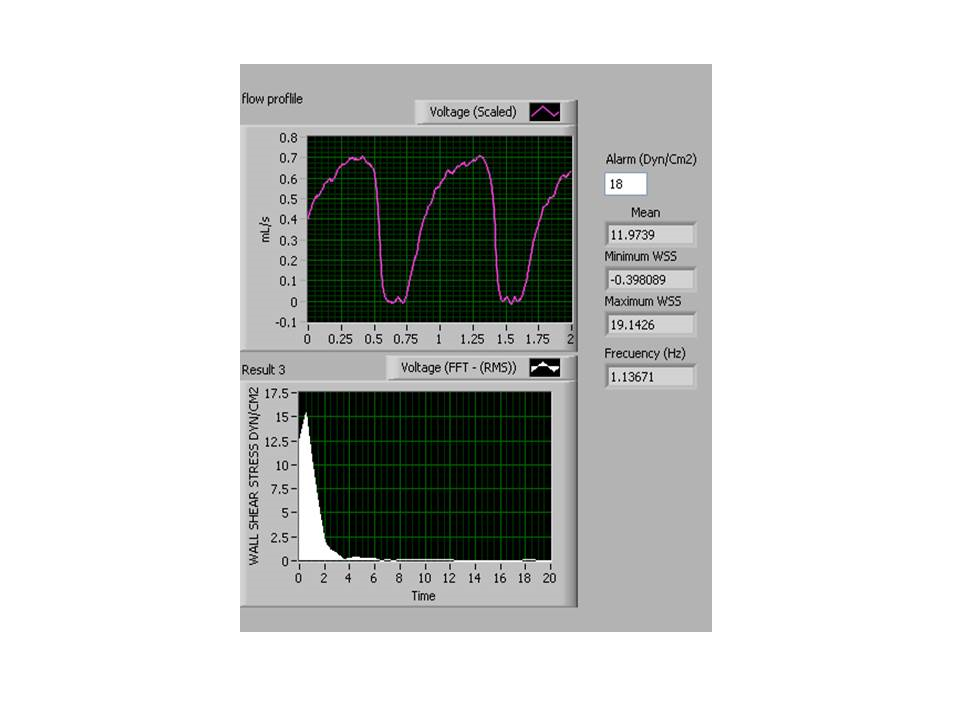

Supplement: S3 Fig — The upper panel of the LabVIEW screen shows a typical flow waveform and the lower panel shows the associated FFT indicating very little contribution from the second or higher harmonics. (TIF) [file pone.0129952.s004.tif]
